# Taxonomic notes on the ground beetles in the genus *Trephionus* Bates, 1883 from central Honshu, Japan (Coleoptera, Carabidae, Sphodrini, Synuchina)

**DOI:** 10.3897/zookeys.742.23752

**Published:** 2018-03-12

**Authors:** Kôji Sasakawa, Hirotarô Itô

**Affiliations:** 1 Laboratory of Zoology, Department of Science Education, Faculty of Education, Chiba University, 1-33 Yayoicho, Inage-ku, Chiba-shi, Chiba, 263-8522 Japan; 2 1-14-16 Awayama, Niigata-shi, Niigata, 950-0843 Japan

**Keywords:** Cryptic species, endophallus, male genitalia, new species, phylogeny, taxonomy

## Abstract

*Trephionus* Bates, 1883, a Japanese endemic genus in the subtribe Synuchina (Coleoptera, Carabidae, Sphodrini), is revised taxonomically based mainly on the shape of the endophallus, a membranous inner sac everted from the aedeagus of the male genitalia. Three known species from central Honshu, *T. kinoshitai* Habu, 1954; *T. shibataianus* Habu, 1978; and *T. babai* Habu, 1978, are re-defined based on this genital character, and five new species are described from the region: *T. cylindriphallus* Sasakawa, **sp**. **n**., *T. niumontanus* Sasakawa, **sp**. **n**., *T. inexpectatus* Sasakawa & Itô, **sp**. **n**., *T. abiba* Sasakawa & Itô, **sp**. **n**., and *T. bifidilobatus* Sasakawa & Itô, **sp**. **n**. The observed interspecies differences in endophallus morphology are discussed in terms of the species-level phylogeny and genus-level taxonomy of *Trephionus*.

## Introduction

*Trephionus* Bates, 1883 is a Japanese endemic genus in the subtribe Synuchina (Coleoptera, Carabidae, Sphodrini) that is distributed in Honshu, Shikoku, and Kyushu (Lindroth 1956; Habu 1978; Casale 1998). This genus is differentiated regionally because of its low dispersal ability due to atrophied hind wings. To date, 15 species-group taxa (14 species and one subspecies) have been described (Habu 1978). However, some of these species are very similar to each other in their external and genital morphology (other than the membranous parts; see below) and the validity of their species statuses remains unresolved (Lindroth 1956). In Carabidae, the taxonomic utility of the shape of the endophallus, a membranous inner sac everted from the aedeagus of the male genitalia, has been demonstrated in studies of various taxa during the last decade. These studies showed that the morphology of the endophallus can provide useful taxonomic information that cannot be obtained from external or other genital morphology. For example, through examination of this genital character, it became apparent that some species include “cryptic species” that cannot be distinguished by their external or traditional genital morphology, but can be distinguished by the endophallus morphology (*e.g.*, Imura 2003; Sasakawa and Kubota 2006a, 2006b). In addition, some externally similar different species, subspecies, or geographical races could not be distinguished by endophallus morphology and were treated as the same species-group taxa (*e.g.*, Sasakawa 2005, 2008; Sasakawa and Kubota 2005). Examination of the endophallus morphology can provide insight into the taxonomy of *Trephionus*, although no studies have examined this genital character in this group.

Our recent research demonstrated that the morphological characteristics of the male endophallus are also taxonomically useful in *Trephionus*. In this study, we revise two species distributed widely in central Honshu: *T. kinoshitai* Habu, 1954 and *T. shibataianus* Habu, 1978. Based on the endophallus morphology, the two species and a related species, *T. babai* Habu, 1978, are re-defined, and five new species are described. The implications of these results for the species-level phylogeny and genus-level taxonomy of *Trephionus* are discussed.

## Materials and methods

**Figure 1.**
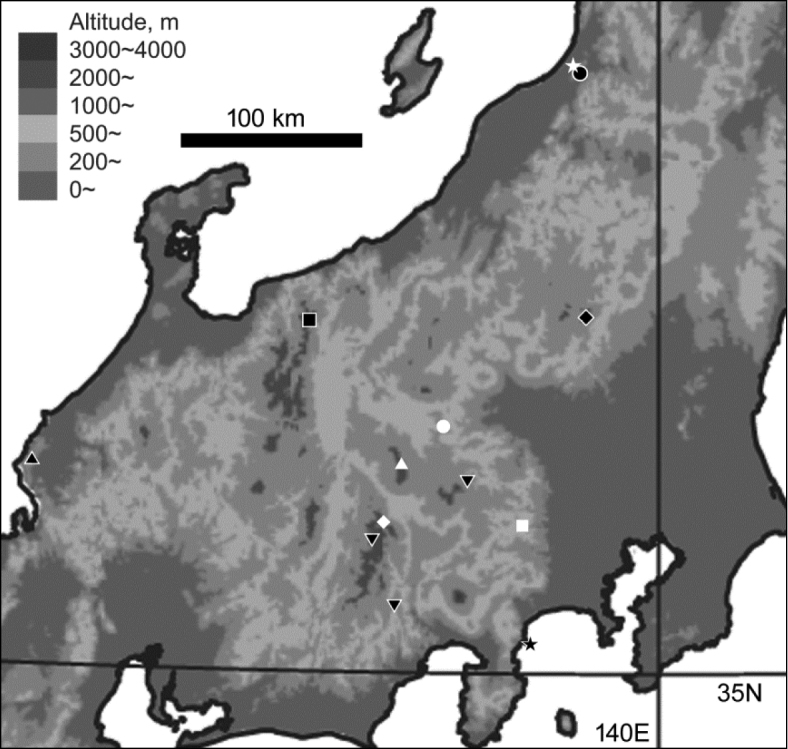
Distribution of *Trephionus* spp. in central Honshu based on specimens with unambiguous identities. ◆ *T. nikkoensis* ★ *T. mikii* ■ *T. kinoshitai* ▼ *T. cylindriphallus* sp. n. ▲ *T. niumontanus* sp. n. ● *T. babai* ○ *T. subcavicola* △ *T. shibataianus* ◇ *T. inexpectatus* sp. n. ☆ *T. abiba* sp. n. □ *T. bifidilobatus* sp. n. The type localities of *T. nikkoensis*, *T. mikii*, and *T. subcavicola*, which were not examined in this study, are shown, although the validities of their species statuses remain to be examined.

Specimens from various localities in central Honshu were examined (Fig. 1). Specimens of the three known species were obtained from the type localities: *T. kinoshitai* from Mt. Shirouma; *T. babai* from the Ishikiri Cave; and *T. shibataianus* from Mt. Naka in the Yatsu Mountains (see specific sections for details). These specimens matched the original descriptions of each species (Habu 1954, 1978) and photographs of the holotypes, which are available in the type-specimen database of the National Agriculture and Food Research Organization (Division of Informatics and Inventory, Insect Systematics Unit, Institute for Agro-Environmental Sciences, National Agriculture and Food Research Organization 2011).

To investigate male genital characters, all male specimens other than a male of *T. kinoshitai* were dissected. The endophallus was everted by injecting toothpaste (White & White; LION, Tokyo, Japan) using an insulin syringe with a pre-attached 29-gauge needle (SS-10M2913; TERUMO, Tokyo, Japan). A new terminology for characters on the endophallus is proposed herein because homologies of most characters between *Trephionus* and other carabid species could not be established. Body length was measured from the mandible apices to the elytral end. To represent the size/shape of some body parts, the following three measurements are defined: (i) the pronotum index (PI) calculated as the width at the widest part divided by the width at the level of postero-marginal setae, which was used by Habu (1978) as a useful diagnostic character, under the name WP/WBP(s); (ii) the basal diameter (BD) of the lobes on the surface of the endophallus in the dorsal view; and (iii) the width of the aedeagus (AW) at the ostium from a dorsal view. The specimens examined are deposited in the collections of the Laboratory of Zoology, Department of Science Education, Faculty of Education, Chiba University, Chiba, Japan (holotypes) and the authors (other specimens).

## Taxonomy

The eight species are similar to each other and share the following adult morphological character states.

**Figures 2–9.**
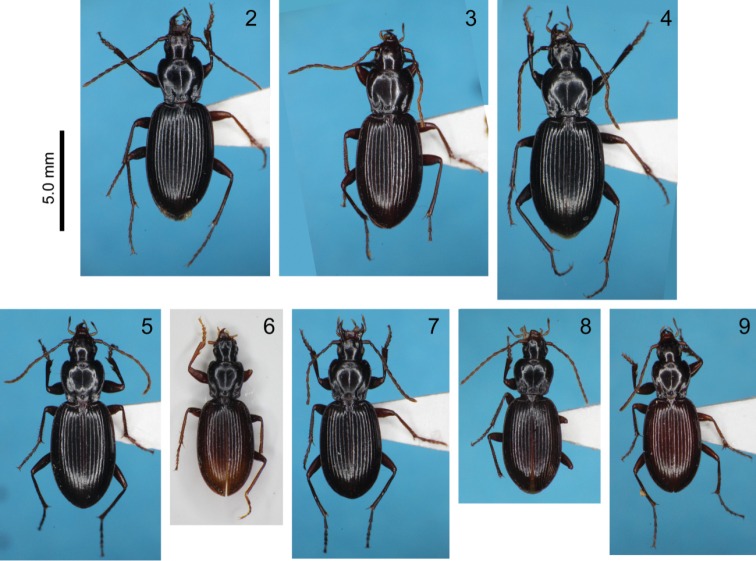
Dorsal view of *Trephionus* species: **2** *T. kinoshitai*, male from Mt. Shirouma **3** *T. cylindriphallus* sp. n., holotype male **4** *T. niumontanus* sp. n., holotype male **5** *T. babai*, male from Mt. Ishikiri **6** *T. shibataianus*, male from Mt. Naka **7** *T. inexpectatus* sp. n., holotype male **8** *T. abiba* sp. n., holotype male **9** *T. bifidilobatus* sp. n., holotype male.

*External characters* (Figs 2–9): Dorsal surface of body reddish brown to black, shiny, not opaque; mouthpart appendages and antennae yellowish to reddish brown; legs light to blackish brown. Hind wings completely absent. Head normal sized, widest at mid-eye level; frontal impressions wide, shallowly depressed, with posterior ends at mid-eye level; surface of frontal impressions more or less wrinkled, without depressed line in the center; frons smooth; two pairs of supraorbital setae, anterior pair at mid eye-level and posterior pair at basal level 1/2 of tempora; eyes less convex, with anteroposterior length slightly longer than that of tempora; tempora not convex, with smooth surface; mentum tooth bifid. Antennae with apices reaching basal level 1/4–1/3 of elytra in male, 1/5–1/4 in female; segments 1–3 and basal 1/4 of segment 4 lack pubescence; segment 2 has 2–3 setae, varying individually.

Pronotum moderately convex, widest at apical 1/4–1/3; anterior margin emarginated, narrower than posterior margin (measured at the level of the postero-marginal setae); anterior angles rounded at apex; lateral margins arcuate except near the base, where roughly sinuate; two marginal setae on each side, anterior setae near widest pronotal point and posterior setae at the posterior end of lateral margins; posterior margin slightly emarginated at median area, arcuate anterolaterally in lateral areas, with the curvature stronger than that of the lateral margins; posterior angles not denticulate; pronotal surface smooth except for posterior margin, which is slightly punctate in some specimens; median line distinct in the middle, but rudimentary or absent near anterior or posterior margins; laterobasal impressions single, wide, moderately concave; both sides of the impressions connected with moderately depressed transverse line.

Elytra oblong, moderately convex; shoulders and apices rounded, not denticulate; intervals barely convex; scutellar stria present, not connected to stria 1; stria 1 connected to stria 2 behind basal margin; one setigerous puncture between the connection of stria 1 and stria 2 and at anterior end of stria 2; interval 3 lacks setigerous punctures. Male sternum 7 lacks sexual characteristics. Legs slender; mid and hind first tarsal segment sulcate outer side in all species, on inner side in some species; tarsal claws smooth inside, not denticulate.

**Figures 10–19.**
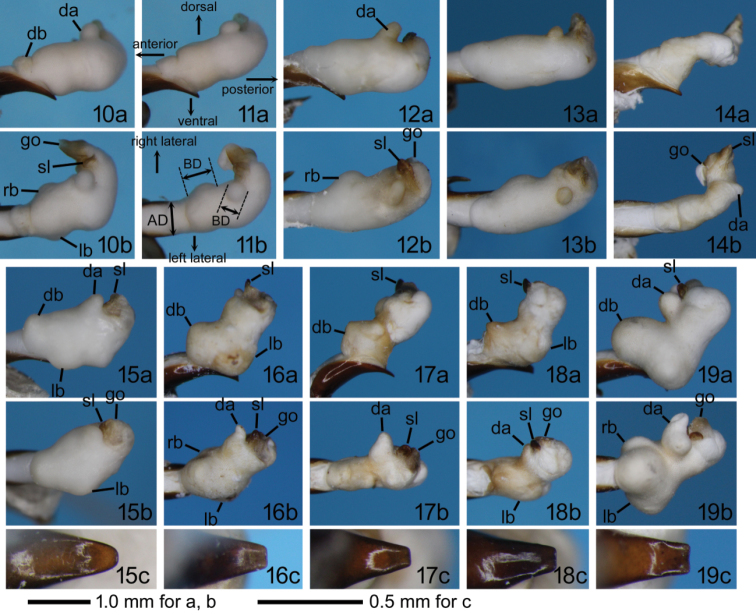
Left lateral (**a**) and dorsal (**b**) views of the endophallus and ventral view of the aedeagal apex (**c**) of *Trephionus* spp.: **10** *T. kinoshitai* from Mt. Shirouma **11** another specimen of *T. kinoshitai* from Mt. Shirouma, showing directions and examples of measurements for descriptions **12** *T. cylindriphallus* sp. n., holotype male; **13** *T. cylindriphallus* sp. n., a paratype male from Abe Pass **14** *T. niumontanus* sp. n., holotype male **15** *T. babai* from Mt. Ishikiri; **16** *T. shibataianus* from Mt. Naka **17** *T. inexpectatus* sp. n., holotype male **18** *T. abiba* sp. n., holotype male **19** *T. bifidilobatus* sp. n., holotype male. Abbreviations: AW, the width of the aedeagus at the ostium from the dorsal view; BD, the basal diameter of the lobe from dorsal view; da, dorsoapical lobe; db, dorsobasal lobe; go, gonopore; lb, left laterobasal lobe; rb, right laterobasal lobe; sl, sclerotized lobe.

*Male genital characters* (Figs 10–19): Aedeagus stout at basal 2/3, narrowed apically at apical 1/3 from lateral view; from dorsal/ventral view, almost parallel-sided from base to ostium, narrowed apically from ostium to apex. Right paramere placoid, sub-oval to sub-square in shape. Left paramere slender, bent at an obtuse angle in the middle. Endophallus with some lobes, unsclerotized one on dorso-subapical or dorso-apical surface (dorsoapical lobe) and sclerotized one between dorsoapical lobe and gonopore (sclerotized lobe) in all species; one on dorso-basal surface (dorsobasal lobe), one on right laterobasal surface (right laterobasal lobe), another on left laterobasal surface (left laterobasal lobe) in some species; gonopore with more or less sclerotized rim.

*Female genital characters*: Gonocoxite 2 digitate and slender, with one ensiform seta on both medial and lateral side; apical nematiform seta absent.

### Trephionus kinoshitai

Taxon classificationAnimaliaColeopteraCarabidae

Habu, 1954

[Japanese name: Shirouma-hoso-hirata-gomimushi] Figs 2 , 10 , 11

Trephionus kinoshitai : Habu (1954): 272, fig. 5 (holotype ♀, “Mt. Ôrenge (Hakuba), Nagano Prefecture” [Mt. Shirouma]); Habu (1978): 397, figs 799, 811, 822, 827, 832 (part); Tanaka (1985): 135, plate 25-fig. 10 (part); Yoshitake et al. (2011): 13 (part); Hovorka and Sciaky (2017): 794.
Synuchus nikkoensis kinoshitai : Lindroth (1956): 526, figs 21B, 22C.

#### Material examined.

5♂1♀, Kamishiro asl. ca. 1084 m, Mt. Shirouma, Hakuba-mura, Nagano Pref. (36.739801°N, 137.813480°E), 7–16.IX.2014 (Pitfall traps baited with 10% acetic acid), K. Sasakawa leg.

#### Diagnosis.

Similar to *T. cylindriphallus* sp. n. and *T. niumontanus* sp. n. in having secondary setae on dorsal side of mid and hind tarsal segment 5, but distinguished from the former by distinctly sinuate lateral margin of pronotum near base and from the latter by smaller body.

#### Description.

Body length: ♂, 8.7–10.2 mm (mean ± SD: 9.5 ± 0.61 mm, n = 5); ♀, 9.0 mm (n = 1). PI: ♂, 1.21–1.29 (mean: 1.27, n = 5); ♀, 1.25 (n = 1). Head and pronotum black; elytra blackish brown to black. Pronotal lateral margins sinuate before hind angles (Fig. 2). Mid and hind tarsal segment 1 bisulcate, segments 5 with two secondary setae on dorsal side. Apex of aedeagus rounded; endophallus (Figs 10, 11) stout, directed posteriorly at basal 2/3, strongly bent right-laterally at apical 1/3; gonopore opening directed right-anterodorsolaterally; dorsobasal lobe slightly swollen; both sides of laterobasal lobe widely swollen, with BD as wide as AW; dorsoapical lobe semi-ellipsoid, with BD half of AW; sclerotization of sclerotized lobe weaker than that of aedeagus; anteroposterior length of sclerotized rim of gonopore twice longer than proximodistal length of sclerotized lobe.

### Trephionus cylindriphallus

Taxon classificationAnimaliaColeopteraCarabidae

Sasakawa sp. n.

http://zoobank.org/254816FE-CBF7-43B4-89EB-A0245546473C

[Japanese name: Chichibu-hoso-hirata-gomimushi] Figs 3 , 12 , 13

#### Type material.

Holotype: ♂, Kawamata, University Forest in Chichibu, The University of Tokyo, Ôtaki, Chichibu-shi, Saitama Pref., 16.X.2009, N. Nishiyama leg. Paratypes: 1♂2♀, same data as the holotype; 1♂3♀, same locality as the holotype (1♂, 17.X.2008, K. Ômura leg.; 1♀, 1.IX.2005, Ômura leg.; 1♀, 6.X.2005, Ômura leg.; 1♀, 10.IX.2009, K. Nishiyama leg.); 1♂, Ôjiro near Abe Pass, Minobu-cho, Yamanashi Pref., 1–2.X.2003, K. Sasakawa leg.; 1♂, Shimakura-rindô, asl. ca. 1000 m, Ôshika-mura, Nagano Pref., 14.IX.1998, M. Hama leg.

#### Diagnosis.

Similar to *T. kinoshitai* and *T. niumontanus* sp. n. in having secondary setae on dorsal side of mid and hind tarsal segment 5, but distinguished from the former by barely sinuate lateral margin of pronotum near base and from the latter by smaller body.

#### Description.

Body length: ♂, 8.9–9.8 mm (mean ± SD: 9.4 ± 0.36 mm, n = 5); ♀, 8.2–10.0 mm (mean ± SD: 9.3 ± 0.84 mm, n = 5). PI: ♂, 1.23–1.25 (mean: 1.24, n = 5); ♀, 1.19–1.24 (mean: 1.22, n = 5). Dorsal surface blackish brown to black. Pronotal lateral margins before hind angles barely sinuate (Fig. 3). Mid and hind tarsal segment 1 bisulcate, segment 5 with two secondary setae on dorsal side. Apex of aedeagus rounded; endophallus (Figs 12, 13) stout, almost straight, directed posteriorly; gonopore opening directed right-dorsolaterally; dorsobasal lobe barely swollen; right laterobasal lobe widely swollen, with BD as wide as AW; left laterobasal lobe absent; dorsoapical lobe semi-ellipsoid, with BD less than half of AW; sclerotization of sclerotized lobe weaker than of aedeagus; anteroposterior length of sclerotized rim of gonopore as long as proximodistal length of sclerotized lobe.

#### Etymology.

The name refers to the robust, cylindrical shape of the endophallus.

### Trephionus niumontanus

Taxon classificationAnimaliaColeopteraCarabidae

Sasakawa sp. n.

http://zoobank.org/78780102-BAFE-412C-A263-8EA48C8D5558

[Japanese name: Inoue-hoso-hirata-gomimushi] Figs 4 , 14

Trephionus kinoshitai : Inoue (2013): 69, fig. 1.

#### Type material.

Holotype: ♂, Mt. Kunimi, asl. ca. 480 m, Fukui-shi, Fukui Pref., 27.VI.2012, S. Inoue leg. Paratypes: 1♀, same data as the holotype; 2♀, Mino-cho, asl. ca. 480 m, Fukui-shi, Fukui Pref., 27.VI.2012, S. Inoue leg.

#### Diagnosis.

Similar to *T. kinoshitai* and *T. cylindriphallus* sp. n. in having secondary setae on dorsal side of mid and hind tarsal segment 5, but distinguished by larger body.

#### Description.

Body length: ♂, 10.6 mm (n = 1); ♀, 10.9 mm (mean ± SD: 10.9 ± 0.03 mm, n = 2). PI: ♂, 1.23 (n = 1); ♀, 1.25–1.26 (n = 2). Dorsal surface black. Pronotal lateral margins sinuate before hind angles (Fig. 4). Mid and hind tarsal segment 1 bisulcate, segment 5 with two secondary setae on dorsal side. Apex of aedeagus rounded; endophallus (Fig. 14) slender, directed posterodorsally at basal 1/3, bent and directed posteriorly at middle 1/3, bent and directed right-laterally at apical 1/3; gonopore opening directed right-anterolaterally; dorsobasal and both sides of laterobasal lobes absent; dorsoapical lobe narrowly swollen, with BD half of AW; sclerotization of sclerotized lobe weaker than of aedeagus; sclerotized rim of gonopore not distinct.

#### Etymology.

The name refers to the Niu Mountains, where the type specimens were collected.

### Trephionus babai

Taxon classificationAnimaliaColeopteraCarabidae

Habu, 1978

[Japanese name: Baba-hoso-hirata-gomimushi] Figs 5 , 15

Trephionus babai : Habu (1978): 404, figs 802, 814, 815, 823, 829, 832, XXXIV-fig. 4 (holotype ♂, “Ishikiri Cave, Nakajô, Niigata Pref.”); Yoshitake et al. (2011): 14; Hovorka and Sciaky (2017): 794.

#### Material examined.

1♂2♀, Mt. Ishikiri (entrance of the Ishikiri Cave), Haguro, Tainai-shi, Niigata Pref. (38.051608°N, 139.435625°E), 7–9.X.2017, H. Itô leg.

#### Diagnosis.

Similar to *T. nikkoensis* in general appearance and the absence of secondary setae on dorsal side of mid and hind tarsal segment 5, but distinguished by wider pronotum and elytral microsculpture, which is isodiametric in *T. babai*, but moderately transverse mesh in *T. nikkoensis* (see Habu, 1978). Distinguished from sympatric *T. abiba* sp. n. by larger body and less sinuate pronotal lateral margin near the base.

#### Description.

Body length: ♂, 9.3 mm (n = 1); ♀, 9.8–10.3 mm (n = 2). PI: ♂, 1.30 (n = 1); ♀, 1.34–1.37 (n = 2). Dorsal surface black. Pronotal lateral margins before hind angles barely sinuate (Fig. 5). Mid and hind tarsal segment 1 bisulcate, segment 5 lacks secondary setae on dorsal side. Apex of aedeagus rounded (Fig. 15c); endophallus (Fig. 15a, b) long oval, directed posteriorly; gonopore opening directed right-dorsolaterally; dorsobasal lobe widely swollen, with BD wider than AW; right laterobasal lobe absent; left laterobasal lobe widely swollen, with BD wider than AW; dorsoapical lobe narrowly swollen, with BD more than half of but less than AW; sclerotization of sclerotized lobe weaker than of aedeagus; sclerotized rim of gonopore not distinct.

### Trephionus shibataianus

Taxon classificationAnimaliaColeopteraCarabidae

Habu, 1978

[Japanese name: Shibata-hoso-hirata-gomimushi] Figs 6 , 16

Trephionus shibataianus : Habu (1978): 405, figs 803, 816, 825, 826, 830, 832 (holotype ♂, “Inago Spa, Nagano Pref.” [the foot of Mt. Naka, the Yatsugatake Mountains]); Tanaka (1985): 135, plate 25-fig. 11 (part); Yoshitake et al. (2011): 14 (part); Hovorka and Sciaky (2017): 794.

#### Material examined.

1♂, Karasawa Spa, Chino-shi, alt. ca. 1900 m (the foot of Mt. Naka, the Yatsugatake Mountains), Nagano Pref., 13–28.VIII.1999, R. Shimoyama leg.

**Diagnosis.** Similar to *T. subcavicola* Uéno and three species to be described below in having truncate apex of aedeagus. Readily distinguished from *T. subcavicola* by the absence of inner sulcus of mid and hind tarsal segment 1 (present in *T. subcavicola*; Habu 1978; Tanaka 1985). Distinguished from the other three species by the smaller PI (i.e., narrower pronotum).

#### Description.

Body length: ♂, 8.4 mm (n = 1). PI: ♂, 1.19 (n = 1). Head and pronotum black; elytra blackish brown to black. Pronotal lateral margins sinuate before hind angles (Fig. 6). Mid and hind tarsal segment 1 sulcate on outer side, not on inner side; tarsal segment 5 lacks secondary setae on dorsal side. Apex of aedeagus truncate (Fig. 16c); endophallus (Fig. 16a, b) stout, almost straight, directed posterodorsally; gonopore opening directed right-dorsoposterolaterally; dorsobasal lobe widely swollen, with BD as wide as AW; both sides of laterobasal lobe widely swollen, with BD as wide as AW; dorsoapical lobe narrowed apically, weakly bent at subapical part, with BD as half of AW; sclerotization of sclerotized lobe the same as aedeagus; sclerotized rim of gonopore indistinct.

### Trephionus inexpectatus

Taxon classificationAnimaliaColeopteraCarabidae

Sasakawa & Itô sp. n.

http://zoobank.org/2BDA8CFE-CF29-4B64-A8FE-9DF92E7E8AF5

[Japanese name: Akaishi-hoso-hirata-gomimushi] Figs 7 , 17

#### Type material.

Holotype: ♂, Kitazawa Pass, alt. ca. 2000 m, Hase-mura, Nagano Pref., 7–15.X.1998, M. Hama leg.

#### Diagnosis.

Readily distinguished from *T. subcavicola* by the absence of inner sulcus of hind tarsal segment 5 (Habu 1978; Tanaka 1985). From other species with truncate aedeagus apex, distinguished by larger body and more blackish dorsal surface (Figs 6–9).

#### Description.

Body length: ♂, 8.8 mm (n = 1). PI: ♂, 1.26 (n = 1). Dorsal surface black. Pronotal lateral margins slightly sinuate before hind angles (Fig. 7). Mid tarsal segment 1 bisulcate; hind tarsal segment 1 sulcate on outer side, not on inner side; mid and hind tarsal segment 5 lacks secondary setae on dorsal side. Apex of aedeagus truncate (Fig. 17c); endophallus (Fig. 17a, b) stout, almost straight, directed posterodorsally; gonopore opening directed right-dorsoposterolaterally; dorsobasal lobe widely swollen, with BD slightly less than half of AW; both sides of laterobasal lobe absent; dorsoapical lobe conical form, widely rounded at apex, with BD more than half of but less than AW; sclerotization of sclerotized lobe the same as aedeagus; anteroposterior length of sclerotized rim of gonopore as long as proximodistal length of sclerotized lobe.

#### Etymology.

The name refers to the unexpected discovery of this species, which is externally similar to other congeneric species with simple aedeagus apex such as *T. kinoshitai*, *T. cylindriphallus* and *T. babai*.

### Trephionus abiba

Taxon classificationAnimaliaColeopteraCarabidae

Sasakawa & Itô sp. n.

http://zoobank.org/0CBC4202-2510-4CC8-8D0F-B86F83F44929

[Japanese name: Ishikiri-hoso-hirata-gomimushi] Figs 8 , 18

#### Type material.

Holotype: ♂, Mt. Ishikiri (entrance of the Ishikiri Cave), Haguro, Tainai-shi, Niigata Pref. (38.051754°N, 139.435703°E), 23.V.2013, H. Itô leg. Paratype: 1♀, same data as the holotype.

#### Diagnosis.

Among the species with truncate aedeagal apex, similar to *T. shibataianus* and *T. bifidilobatus* sp. n. in having small body. Distinguished from the former by larger PI (i.e., wider pronotum) and from the latter by blacker dorsal surface (Figs 8, 9). Readily distinguished from sympatric *T. babai* by smaller body and distinctly sinuate pronotal lateral margin near the base.

#### Description.

Body length: ♂, 8.1 mm (n = 1); ♀, 9.8 mm (n = 1). PI: ♂, 1.27 (n = 1); ♀, 1.31 (n = 1). Head and pronotum black; elytra blackish brown to black (Fig. 8). Mid and hind tarsal segment 1 biculcate, segment 5 without secondary setae on dorsal side. Apex of aedeagus truncate (Fig. 18c); endophallus (Fig. 18a, b) stout, almost straight, directed posterodorsally; gonopore opening directed right-dorsoposterolaterally; dorsobasal lobe narrowly swollen, with BD less than AW; right laterobasal lobe absent; left laterobasal lobe widely swollen, with BD as wide as AW; dorsoapical lobe simple form, rudimentary, with BD less than half of AW; sclerotization of sclerotized lobe as same as aedeagus; sclerotized rim of gonopore not distinct.

#### Etymology.

The name is an anagram of the specific name of the sympatric species *T. babai*.

### Trephionus bifidilobatus

Taxon classificationAnimaliaColeopteraCarabidae

Sasakawa & Itô sp. n.

http://zoobank.org/5B16A804-AF56-4C34-A1F5-B66A42CA6A44

[Japanese name: Mitake-hoso-hirata-gomimushi] Figs 9 , 19

#### Type material.

Holotype: ♂, Kotosawa, alt. ca. 310 m, Mitake, Oume-shi, Tokyo (35.795931°N, 139.168746°E), 10–12.IX.2009, K. Sasakawa leg.

**Diagnosis.** Similar to *T. shibataianus* and *T. abiba* sp. n. in having truncate aedeagal apex and small body, but distinguished by larger PI (i.e., wider pronotum) and reddish brown elytra.

#### Description.

Body length: ♂, 8.2 mm (n = 1). PI: ♂, 1.32 (n = 1). Head and pronotum black, elytra reddish brown. Pronotal lateral margins sinuate before hind angles (Fig. 9). Mid tarsal segment 1 bisulcate; hind tarsal segment 1 sulcate on outer side, not on inner side; tarsal segment 5 lacks secondary setae on dorsal side. Apex of aedeagus truncate, slightly emarginated at middle (Fig. 19c); endophallus (Fig. 19a, b) stout, almost straight, directed posterodorsally; gonopore opening directed right-dorsoposterolaterally; dorsobasal lobe widely swollen, with BD wider than AW; right laterobasal lobe widely swollen, with BD as wide as AW; left laterobasal lobe widely swollen, with BD wider than AW; dorsoapical lobe markedly developed, bifid at apex, with BD more than 0.8 times AW; sclerotization of sclerotized lobe as same as aedeagus; anteroposterior length of sclerotized rim of gonopore as long as proximodistal length of sclerotized lobe.

#### Etymology.

The name refers to the bifid apex of the dorsoapical lobe of the endophallus.

## Discussion

This study revealed that *Trephionus* has diversified in terms of the male endophallus and there are “cryptic species” that can be distinguished only by the endophallus. These findings indicate the need for re-definition of the known *Trephionus* species based on this genital morphology. To this end, new specimens from the type localities will be required for most species because the dissection of the membranous parts of the genitalia, such as the endophallus, is usually difficult in old specimens and because the type specimens of all of the known *Trephionus* were collected over 40 years ago (Habu 1978). Re-definition of the known species may lead to the discovery of additional cryptic species. In this study, some specimens that were identified as *T. kinoshitai* or *T. shibataianus* in the previous *Trephionus* taxonomy (Habu 1978) have been determined to be different species through comparative endophallus morphology. Similar results may be observed in other known species, especially in species with a wide distribution (e.g., *T. nikkoensis*). Future studies are required.

**Figure 20.**
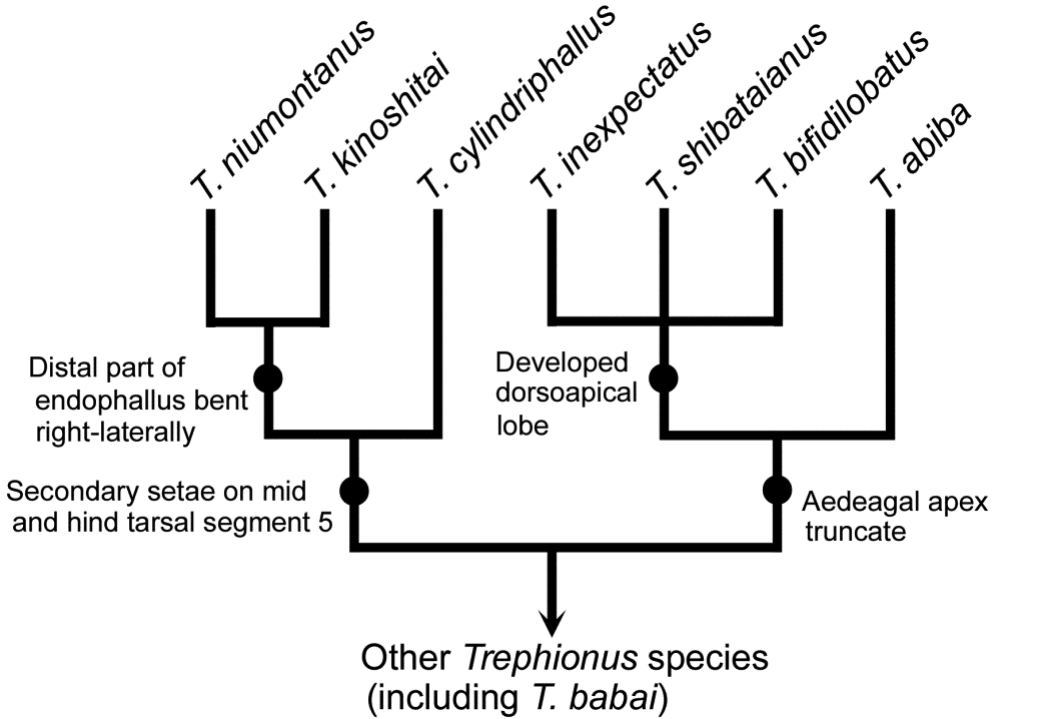
Phylogenetic relationships of the eight *Trephionus* species and their synapomorphies.

Interspecies differences in endophallus morphology and distribution give insights into the phylogeny and differentiation of *Trephionus*. Of the eight species treated here, *T. kinoshitai*, *T. cylindriphallus*, and *T. niumontanus* are considered to be closely related because they share a character that is found only in a few species of Synuchina, *i.e.*, secondary setae on the dorsal side of the mid and hind last tarsal segments (Habu 1978). Of the three species, *T. kinoshitai* and *T. niumontanus* have an endophallus in which the distal part is strongly bent (Figs 10b, 11b, 14b); this character state has been reported only in these species within the subtribe and is considered a synapomorphy uniting the two species. Among the remaining five species, *T. shibataianus*, *T. inexpectatus*, *T. abiba*, and *T. bifidilobatus* are undoubtedly closely related because they share a truncate aedeagal apex (Figs 16c, 17c, 18c, 19c), which is found only in these species within Synuchina and is an unambiguous apomorphic character (Lindroth 1956; Habu 1978). Of the four species, *T. shibataianus*, *T. inexpectatus*, and *T. bifidilobatus* probably form a clade because they have evolved a dorsoapical lobe with a complicated shaped (compared with *T. abiba*). Note that in both clades, the western species are more derived than the eastern species (Figs 1, 20). This pattern agrees with the results of the comparative external morphology of all of the known species of *Trephionus*. In all but one species distributed in the area east of Kinki, the mid first tarsal segment is sulcate on both the outer and inner sides. However, in all of the species from the more western area, there is no sulcus on the inner side (Habu 1978). Because the absence of the inner sulcus is limited to a few species of Synuchina (Lindroth 1956; Habu 1978), it is considered an apomorphic character state. Moreover, some species from the western area are depigmented and are derivative in terms of body coloration (Habu 1978). One possible explanation for these results is that the ancestors of *Trephionus*, which have no apomorphic character, were distributed in the eastern part of the current distribution of *Trephionus*, probably in an area east of central Honshu, and that descendants with the apomorphic character(s) dispersed westward. To test this assumption, re-examination of the species phylogeny, including species from the western area, is required.

Our results also provide insights into the genus-level taxonomy. Despite the marked morphological diversification, the endophallus of all of the species examined has two lobes in common: the dorsoapical and sclerotized lobes. Regarding the dorsoapical lobe, a similar lobe has been reported in some groups of Carabidae (*e.g.*, Sasakawa et al. 2006; Sasakawa 2016), although their homology is unclear. Regarding the sclerotized lobe, however, no similar character has been reported in other members of Carabidae, and it appears to be an autapomorphy of *Trephionus*. Future studies should examine the endophallus of *T. nikkoensis*, the type species of the genus, and that of genera potentially related to *Trephionus*. These results will provide valuable information not only for re-definition of *Trephionus* but also regarding the importance of the endophallus in the genus-level taxonomy of Synuchina.

## Supplementary Material

XML Treatment for Trephionus kinoshitai

XML Treatment for Trephionus cylindriphallus

XML Treatment for Trephionus niumontanus

XML Treatment for Trephionus babai

XML Treatment for Trephionus shibataianus

XML Treatment for Trephionus inexpectatus

XML Treatment for Trephionus abiba

XML Treatment for Trephionus bifidilobatus
